# Association of Age of Metabolic Syndrome Onset With Cardiovascular Diseases: The Kailuan Study

**DOI:** 10.3389/fendo.2022.857985

**Published:** 2022-03-17

**Authors:** Zegui Huang, Xianxuan Wang, Xiong Ding, Zefeng Cai, Weijian Li, Zekai Chen, Wei Fang, Zhiwei Cai, Yulong Lan, Guanzhi Chen, Weiqiang Wu, Zhichao Chen, Shouling Wu, Youren Chen

**Affiliations:** ^1^ Department of Cardiology, Second Affiliated Hospital of Shantou University Medical College, Shantou, China; ^2^ School of Public Health, North China University of Science and Technology, Tangshan, China; ^3^ Department of Epidemiology, University Medical Center Groningen, University of Groningen, Groningen, Netherlands; ^4^ Second Clinical College, China Medical University, Shenyang, China; ^5^ Department of Cardiology, Kailuan General Hospital, Tangshan, China

**Keywords:** metabolic syndrome, onset age, cardiovascular diseases, myocardial infarction, strokes

## Abstract

**Background:**

Metabolic syndrome (MetS) is associated with an increased risk of incident cardiovascular diseases (CVD), but the association between the new-onset MetS at different ages and the CVD risk remain unclear.

**Methods:**

This was a prospective study comprising a total of 72,986 participants without MetS and CVD who participated in the Kailuan study baseline survey (July 2006 to October 2007). All participants received the biennial follow-up visit until December 31, 2019. In addition, 26,411 patients with new-onset MetS were identified from follow-up, and one control participant was randomly selected for each of them as a match for age ( ± 1 year) and sex. In the end, a total of 25,125 case-control pairs were involved. Moreover, the Cox proportional hazard model was established to calculate the hazard ratios (HR) for incident CVD across the onset age groups.

**Results:**

According to the median follow-up for 8.47 years, 2,319 cases of incident CVD occurred. As MetS onset age increased, CVD hazards gradually decreased after adjusting for potential confounders. Compared with non-MetS controls, the HR and the 95% confidence interval (CI) for CVD were 1.84 (1.31–2.57) in the MetS onset age <45 years group, 1.67 (1.42–1.95) for the 45–54 years group, 1.36 (1.18–1.58) for the 55–64 years group, and 1.28 (1.10–1.50) for the ≥65 years group, respectively (p for interaction = 0.03).

**Conclusions:**

The relative risks of CVD differed across MetS onset age groups, and the associations was more intense in the MetS onset group at a younger age.

## Introduction

Metabolic syndrome (MetS) indicates the clustering of metabolic abnormalities such as abdominal obesity, dyslipidemia, hyperglycemia, and hypertension which relate to an enhanced risk of cardiovascular diseases (CVD) (1). According to estimates, approximately 20%–25% of adults suffer from MetS worldwide, with the incidence and prevalence still surging. Thus, it is a critical global public health challenge (2). It was estimated that China, as the largest developing country, has 450 million adults with MetS (3) and the prevalence of MetS among the young population doubled between 2002 and 2012 (4). Moreover, in developed countries, such as among American adults, the prevalence of MetS grew from 32.5% in 2011 to 36.9% in 2016. In these years, MetS prevalence in the young population aged 20–39 years surged significantly (from 16.2% to 21.3%) at a faster rate than that in older individuals (5). Furthermore, previous studies have proved that CVD risk factor exposure in early life increases the risk of adverse outcomes more than late-life exposure (6, 7).

The Uppsala Longitudinal Study of Adult Men (ULSAM) study demonstrated that individuals with younger MetS were featured with a higher relative risk of CVD, and the relative risk lowered as prevalent age increased (8); however, its limitation was that the MetS onset age was not determined. In addition, recent studies have revealed that among the components of the MetS, new-onset hypertension (9) or diabetes (10, 11) at a younger age is more strongly associated with the CVD risk. Therefore, it was assumed that as a multi-component combination, the new-onset MetS at a younger age was characterized with a higher relative risk with CVD, when compared with older individuals. To date, the relationship between new-onset MetS at different ages and CVD risk is still unknown. Accordingly, we used data in the Kailuan Study (Registration Number: CHICTR-TNRC-11001489) to explore the association between MetS onset age with CVD.

## Methods

### Study Participants

The Kailuan study is an ongoing prospective cohort study performed in Tangshan, China. Moreover, the design and procedure details of the study have been described previously (12, 13). In July 2006–October 2007, a total of 101,510 adult participants in the employee and retiree populations of the Kailuan Group were enrolled in the baseline survey. Then, they were followed up every two years to undergo health assessments and questionnaires on demographic features, lifestyle factors, and medication history. In the current study, we excluded individuals with missing diagnosis data of MetS, being diagnosed of MetS at baseline or with myocardial infarction (MI) or strokes before the diagnosis of MetS. In addition, it should be mentioned that all the involved participants were followed up till December 31, 2019. The study was conducted according to the principles of the Declaration of Helsinki and was approved by the ethics committee of the Kailuan General Hospital. Written informed consent was obtained from all participants.

Of the 72,986 participants remained eligible in the baseline survey (2006–2007), 26,411 were confirmed with MetS until December 31, 2017 (the sixth survey). The MetS onset date was defined as the date of the examination at which MetS was first diagnosed. As for the control group participants, they were selected from non-MetS participants involved in the examination of the same year as the case was determined and matched for age ( ± 1 years) and sex. In addition, the follow-up period was initiated with the diagnosis date for participants with MetS and with the same year for the matched control participants. For example, a new-onset of MetS was first identified in a 60-years-old male participant in 2010, and the matched control was chosen randomly from the male participant aged 59–61 years, who was not diagnosed with the MetS in follow-up and received examination in 2010 as well, both were monitored since 2010. Finally, 25,125 non-MetS participants and 25,125 new-onset MetS participants were incorporated into this study (**Figure S1**).

### Definition of New-Onset MetS

The diagnosis of the MetS follows the definition of the International Diabetes Federation (14): Central obesity, as evaluated using waist circumference (WC), was considered significant, defined as WC≥90 cm for men and WC≥80 cm for women; with two or more of the four factors below: increased triglycerides (>1.7 mmol/L), decreased high-density lipoprotein cholesterol (HDL-C) (<1.03 mmol/L for men and <1.29 mmol/L for women), increased blood pressure (systolic ≥130 mmHg or diastolic≥85 mmHg or treatment for formerly diagnosed hypertension), and increased fasting plasma glucose (FPG) (≥5.6 mmol/L or formerly diagnosed type 2 diabetes). Additionally, new-onset MetS were defined as participants with prior non-MetS being diagnosed with MetS during a follow-up visit.

### Measurement of WC, Blood Pressure, and Biochemical Parameters

The participant acquired a vertical standing posture, with feet approximately 30 cm apart naturally; therefore, the weight was evenly distributed with stable breathing. Following this, an inelastic soft ruler was used to horizontally measure the abdomen around the midpoint of the line between the anterior superior iliac spine and the lower edge of the 12th rib. Furthermore, the measurement was performed against the skin and accurate to 0.1 mm. In addition, all participants were instructed to refrain from smoking and drinking tea or coffee 30 min before blood pressure (BP) measurement. After preparation, BP was calculated on the left upper arm using the calibrated mercury sphygmomanometer and, after a 5-min rest, measured again. If the difference between the two measurements was ≥5 mmHg, the BP was measured again, and the average value in blood pressure measurements was finally recorded. Furthermore, the participant fasted overnight before the examination, and 5 mL of venous blood was collected from the middle of the elbow on an empty stomach on the morning of the physical examination day. FPG, triglycerides, HDL-C, and high-sensitivity C-reactive protein (hs-CRP) were calculated by examiners using the Hitachi 7080 automatic biochemical analyzer.

### Outcome Variables: Cardiovascular Disease and Its Subgroup

The outcome of the study was the first occurrence of CVD. CVD types comprised MI and strokes. Following this, the ICD-10th revision code was used to identify cases of CVD (15, 16). Data on CVD diagnoses were collected from 11 hospital discharge registers and municipal social insurance institutions of the Kailuan Group and upgraded annually at follow-up. Moreover, a panel of three experienced physicians collected and reviewed annual discharge records; thus, determining suspected CVD cases. In addition, the diagnosis of MI was determined according to the clinical symptoms, electrocardiogram, and dynamic changes of cardiac enzymes of patients (17). Moreover, the diagnosis of stroke is based on neurological signs and symptoms, combined with imaging examinations such as computed tomography or magnetic resonance imaging (18), which could be classified into two subtypes, namely the ischemic stroke (IS) and hemorrhagic stroke (HS).

### Definitions of Covariate

Hypertension was defined as (1) SBP ≥140 mmHg and diastolic blood pressure (DBP) ≥90 mmHg, or (2) having a history of clearly diagnosed hypertension or taking antihypertensive drugs. Whereas, the definition for diabetes mellitus was (1) FBG ≥7.0 mmol/L, or (2) having a history of clearly diagnosed diabetes or taking hypoglycemic drugs. In addition, physical exercise was defined as exercise ≥3 times a week, with the duration of each exercise at least 30 min, while ever-smokers were defined as participants with a smoking history or smoking currently, and ever-drinkers referred to those with a drinking history or drinking currently. In addition, as for family history for CVD, a history of MI or strokes in one of the parents was defined.

### Statistical Analysis

Patients with new-onset MetS and matched control groups according to their age of onset were divided into: <45 years, 45–54 years, 55–64 years, and ≥65 years groups. Continuous variables were contrasted by analysis of variance or the Kruskal-Wallis test according to distribution and categoric variables were analyzed by conducting the chi-square test. The incidence density was measured by dividing the number of events by the total person-years of follow-up (1000 per person-year). Additionally, the Cox proportional hazard models taking age as the fundamental time scale were established to analyze the HR and 95% CI of CVD (MI, IS, and HS) for new-onset MetS in comparison with the non-MetS, while the multivariate-adjusted models were adjusted by heart rate (HR), hs-CRP, ever-smokers (yes or no), ever-drinkers (yes or no), physical activity (yes or no), education level, and family history of CVD (yes or no). Moreover, multiple imputations by fully conditional specification were adopted to impute the missing value of covariates (19).

For verifying research robustness, some sensitivity analyses were performed. Firstly, considering different definitions of the MetS might have influenced the results, the MetS defined by CDS (20), NCEP (21) or JIS (22) was utilized for the study. Secondly, to minimize reverse causality, outcome events in the first year during follow-up were excluded. Thirdly, taking account of the effect of treatment on the outcome, the participants who received medical treatment (the use of anti-hypertensive drugs, anti-diabetic drugs or lipid-lowering drugs) were excluded. Finally, the incident CVD analysis was repeated with the Fine-Gray model, considering death from non-CVD as a competing risk. *p*<0.05 (two-sided test) was considered statistically significant. We used SAS 9.4 (SAS Institute, Inc, Cary, NC) software for statistical analysis.

## Results

### Baseline Characteristics

The baseline characteristics of participants can be seen in **Tables 1** and **2**. Among 25,125 non-MetS and 25,125 new-onset MetS participants, 81.93% were men, and the mean onset age of MetS was 54.04 ± 11.41 years. The participants were classified into the following groups by age: 10,250 at an age of <45 years, 16,298 at an age of 45–54 years, 15,054 at an age of 55–64 years, and 8,108 at an age of ≥65 years. Compared with the non-MetS control group, participants with the new-onset MetS have a greater likelihood of being ever-smokers or ever-drinkers; with a higher WC, HR, SBP, DBP, triglycerides, FPG, and hs-CRP level, and being featured with higher prevalence for hypertension, diabetes, and family history for CVD; however, the HDL-C and education level was lower (**Table 1**). Participants with younger-onset MetS had higher TG and HR levels and tended to be ever-smokers, ever-drinkers, and physically inactive. These individuals had a higher percentage of CVD family history, while featured lower SBP, FPG, and hs-CRP level and lower prevalence of hypertension, diabetes, and the proportion of medical treatment compared with those who had an older onset age (**Table 2**). Comparisons between groups had statistical significance (*p*<0.05).

**Table 1:** Basic characteristics for participants with new-onset MetS and their non-MetS controls.

Variables	Non-MetS (n=25125)	New-Onset MetS (n=25125)	P value
Age, years	54.04 ± 11.41	54.04 ± 11.41	—
Male n (%)	20584 (81.93)	20584 (81.93)	—
Waist circumference, cm	83.06 ± 8.48	94.32 ± 7.29	<0.01
Heart rate, beats/min	73.32 ± 10.54	75.36 ± 10.84	<0.01
Systolic blood pressure, mm/Hg	127.77 ± 19.05	138.87 ± 18.04	<0.01
Diastolic blood pressure, mm/Hg	81.26 ± 10.53	87.25 ± 10.52	<0.01
Triglycerides, mmol/l	1.05 (0.77,1.41)	1.74 (1.12,2.40)	<0.01
HDL-C, mmol/l	1.56 ± 0.47	1.39 ± 0.46	<0.01
FPG, mmol/l	5.31 ± 1.21	6.04 ± 2.21	<0.01
hs-CRP, mmol/l	1.00 (0.46, 2.30)	1.30 (0.60, 2.95)	<0.01
Hypertension n (%)	8195 (32.62)	15224 (60.59)	<0.01
Diabetes mellitus n (%)	1152 (4.59)	3425 (13.63)	<0.01
Anti-hypertensive drugs n (%)	4555 (18.13)	6840 (27.22)	<0.01
Anti-diabetic drugs n (%)	411 (1.64)	1264 (5.03)	<0.01
Lipid-lowering drugs n (%)	294 (1.17)	587 (2.34)	<0.01
Family history of CVD n (%)	2982 (11.87)	3248 (12.93)	<0.01
Ever-smokers n (%)	10085 (40.14)	10340 (41.15)	0.02
Ever-drinkers n (%)	10907 (43.41)	11655 (46.39)	<0.01
Physical exercise n (%)	4313 (17.17)	4604 (18.32)	<0.01
Education			<0.01
High school n (%)	21454 (85.39)	21593 (85.94)	
College or above n (%)	1708 (6.80)	1499 (5.97)	

Data are presented as mean ± SD, n (%), or median (P_25_,P_75_); Characteristics were assessed in the examination cycle when new-onset MetS was first identified.

HDL-C, high density lipoprotein cholesterol; FPG, fasting plasma glucose; hs-CRP, high sensitivity C-reactive protein.

**Table 2:** Basic characteristics for new-onset MetS participants across age groups.

Variables	<45 Y(n=5125)	45~54 Y (n=8419)	55~64 Y (n=7527)	≥65 Y (n=4054)	P Value
Age, years	37.95 ± 5.35	50.53 ± 2.90	59.54 ± 2.77	71.48 ± 5.13	<0.01
Male n (%)	4328 (84.45)	6312 (74.97)	6284 (83.49)	3660 (90.28)	<0.01
Waist circumference, cm	94.43 ± 7.19	93.49 ± 7.42	94.56 ± 7.11	95.44 ± 7.26	<0.01
Heart rate, beats/min	76.12 ± 10.56	75.63 ± 10.59	74.72 ± 10.83	74.93 ± 11.60	<0.01
Systolic blood pressure, mm/Hg	130.75 ± 14.20	136.10 ± 16.84	142.25 ± 18.06	148.62 ± 18.73	<0.01
Diastolic blood pressure, mm/Hg	86.65 ± 9.91	88.16 ± 10.59	87.77 ± 10.58	85.16 ± 10.71	<0.01
Triglycerides, mmol/l	2.07 (1.45, 2.91)	1.83 (1.20, 2.57)	1.58 (1.06, 2.17)	1.34 (0.93,1.89)	<0.01
HDL-C, mmol/l	1.35 ± 0.45	1.40 ± 0.45	1.40 ± 0.46	1.41 ± 0.47	<0.01
FPG, mmol/l	5.65 ± 1.52	6.02 ± 2.17	6.21 ± 2.62	6.24 ± 2.10	<0.01
hs-CRP, mmol/l	1.30 (0.61, 2.80)	1.30 (0.60, 2.82)	1.30 (0.59, 3.00)	1.50 (0.63, 3.40)	<0.01
Hypertension n (%)	2357 (45.99)	4829 (57.36)	5031 (66.84)	3007 (74.17)	<0.01
Diabetes mellitus n (%)	385 (7.51)	1126 (13.37)	1217 (16.17)	697 (17.19)	<0.01
Anti-hypertensive drugs n (%)	743 (14.50)	1955 (23.22)	2477 (32.91)	1665 (41.07)	<0.01
Anti-diabetic drugs n (%)	124 (2.42)	403 (4.79)	491 (6.52)	246 (6.07)	<0.01
Lipid-lowering drugs n (%)	71 (1.39)	196 (2.33)	218 (2.90)	102 (2.52)	<0.01
Family history of CVD n (%)	682 (13.31)	1290 (15.32)	956 (12.70)	320 (7.89)	<0.01
Ever-smokers n (%)	2633 (51.38)	3815 (45.31)	2824 (37.52)	1068 (26.34)	<0.01
Ever-drinkers n (%)	3043 (59.38)	4113 (48.85)	3072 (40.81)	1427 (35.20)	<0.01
Physical exercise n (%)	590 (11.51)	1223 (14.53)	1750 (23.25)	1041 (25.68)	<0.01
Education					<0.01
High school n (%)	4290 (83.71)	7703 (91.50)	6600 (87.68)	3000 (74.00)	
College or above n (%)	770 (15.02)	379 (4.50)	204 (2.71)	146 (3.60)	

Data are presented as mean ± SD, n (%), or median (P_25_,P_75_); Characteristics were assessed in the examination cycle when new-onset MetS was first identified.

HDL-C, high density lipoprotein cholesterol; FPG, fasting plasma glucose; hs-CRP, high sensitivity C-reactive protein.

### Associations of the MetS Onset Age With CVD and Its Subgroup

**Figure 1:**
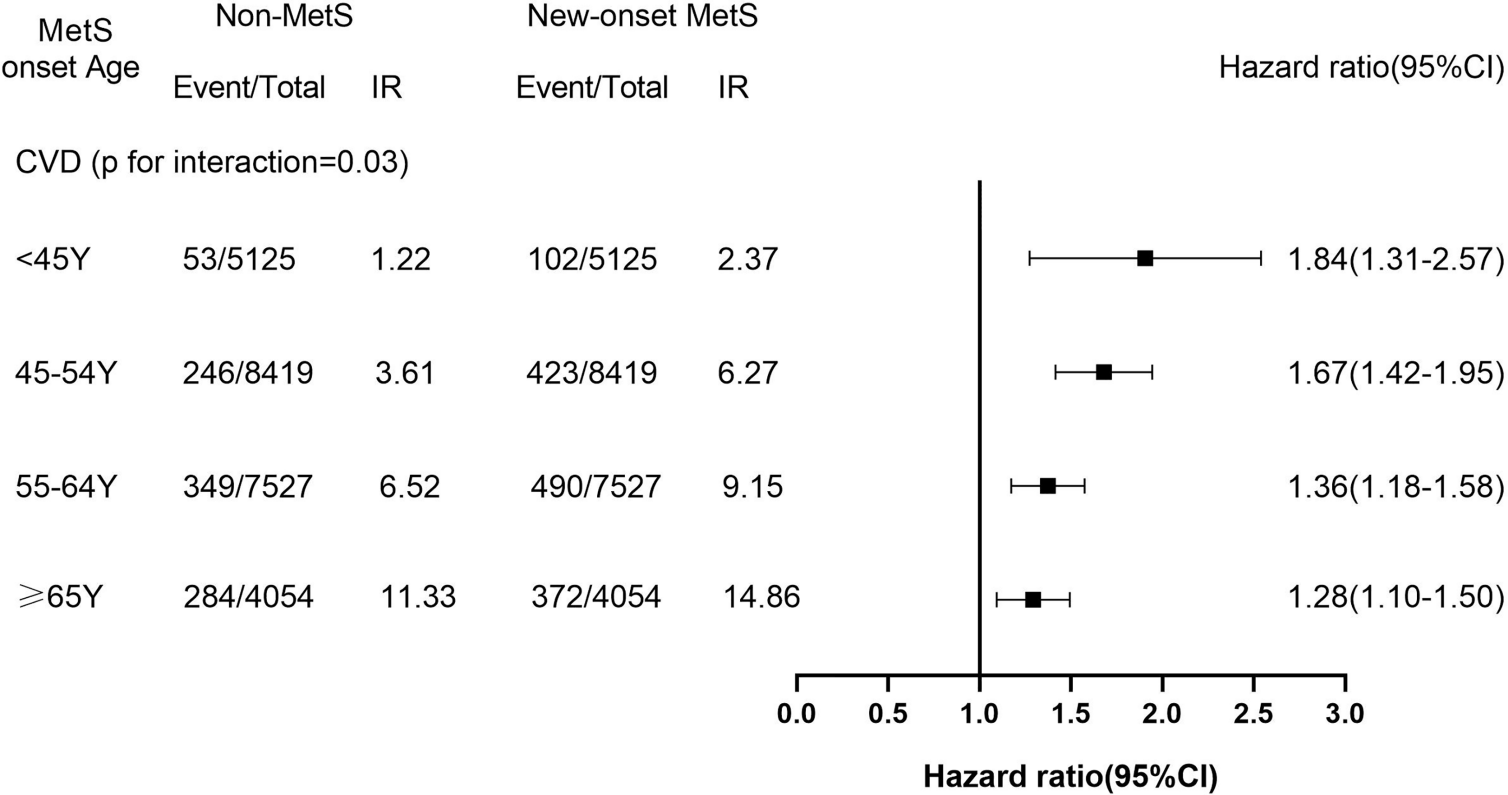
The Hazard Ratios (95%CI) of Incident Cardiovascular Disease of New-Onset MetS Participants Across Age Group. IR, incidence rate (per 1000 person-years). The models were adjusted for HR, hs-CRP, ever-smokers, ever-drinkers, physical exercise, education and family history of cardiovascular disease. CI, confidence interval.

**Figure 2:**
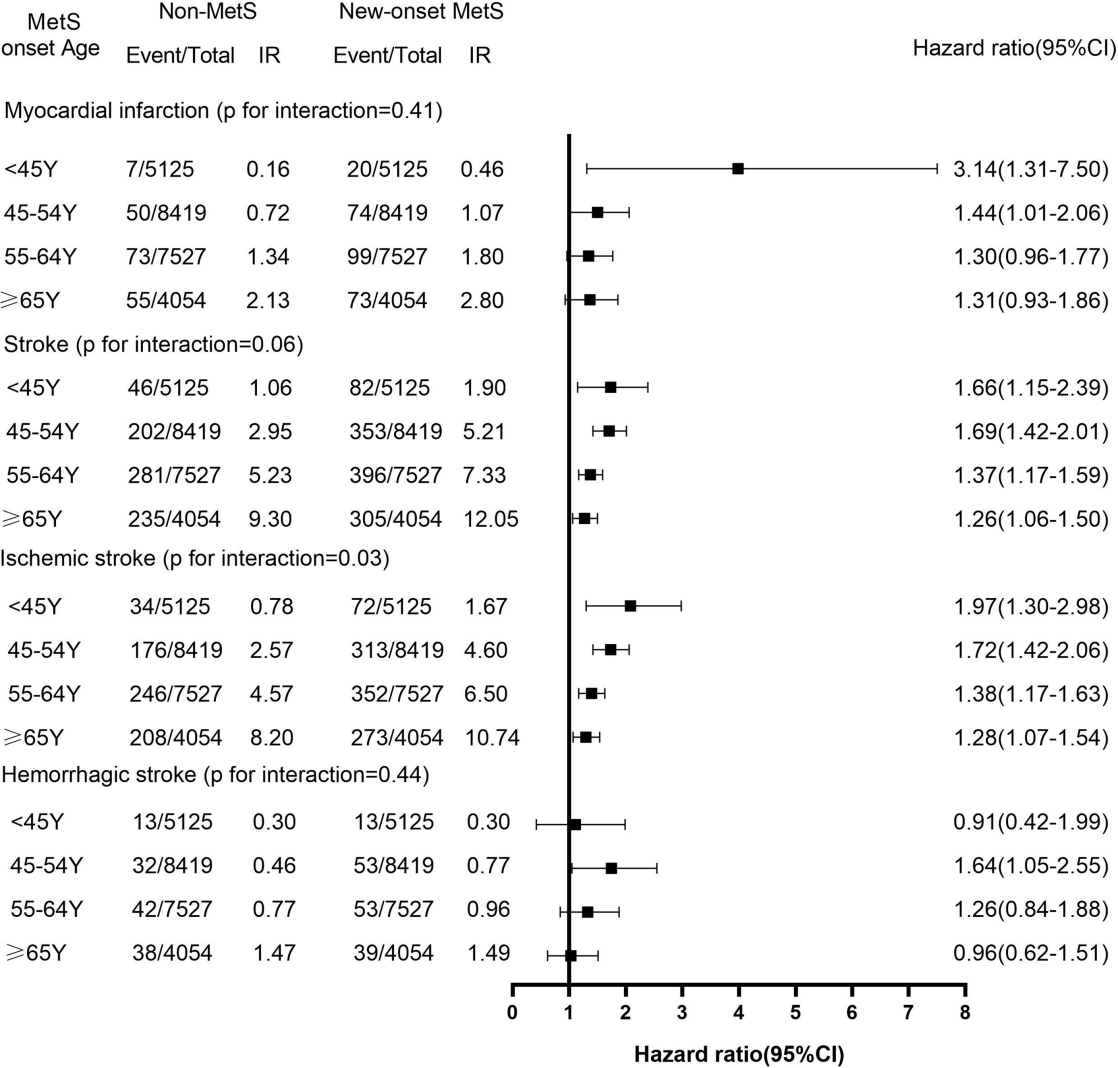
The Hazard Ratios (95%CI) of the Subtypes of Cardiovascular Disease of New-Onset MetS Participants Across Age Group. IR, incidence rate (per 1000 person-years). The models were adjusted for HR, hs-CRP, ever-smokers, ever-drinkers, physical exercise, education and family history of cardiovascular disease. CI, confidence interval.

Through a median follow-up for 8.47 years, 2,319 cases with incident CVD were recognized (451 with MI, 1,674 with IS, and 283 with HS). The incidence density of CVD and its subgroup is illustrated in **Figure 1** and **Figure 2**. After adjustment for potential confounders, as MetS onset age increased, CVD hazards were progressively weakened. Compared with non-MetS controls, the HR and 95% CI for CVD, MI, and strokes were1.84 (1.31–2.57), 3.14 (1.31–7.50) and 1.66 (1.15–2.39) for the MetS onset age <45 years group, 1.67 (1.42–1.95), 1.44 (1.01–2.06) and 1.69 (1.42–2.01) for the 45–54 years group, 1.36 (1.18–1.58), 1.30 (0.96– 1.77) and 1.37 (1.17–1.59) for the 55–64 years group, and 1.28 (1.10–1.50), 1.31 (0.93–1.86) and 1.26 (1.06–1.50) for the≥65 years group, respectively. In the subtypes of strokes, the outcome trend of IS was similar to that of CVD, while the results of HS were mostly not statistically significant.

### Sensitivity Analyses

The results for MetS defined by CDS, NCEP or JIS were similar to the main analysis (**Table S1**). The results of excluding outcome events occurring within the first year of follow-up or received medical treatment respectively also were consistent with the main analysis (**Table S2** and **Table S3**). Taking into account the competitive risk of non-cardiovascular death, we used the Fine-Gray model to assess the association of new-onset MetS at different ages group with CVD, the result still was steady (**Table S4**).

## Discussion

The important finding of this study was that new-onset MetS of all ages are related to an increase in relative risk of CVD, and the risk is age-dependent. Alternatively, the relative risk of the MetS and CVD attenuates with increasing onset age. Similar patterns were found in subtypes of CVD, and the results remained robust among sensitivity analyses. The results demonstrate individuals having a younger MetS onset age had a higher relative risk of incident CVD.

To the best of our knowledge, merely a few studies have examined the association of the MetS with CVD risk in individuals of different ages. ULSAM study had found that the association of the MetS with CVD risk reduced with the increasing age; and the risk ratio and 95% CI of the MetS and CVD risk dropped from 2.77 (1.90–4.05) at 50 years of age to 1.30 (1.05–1.60) at 82 years (8). However, as they did not consider the diagnosis time of MetS, it was impossible to determine the exposure time of MetS before observation began. Whereas our study reported that the relative risk of the MetS and CVD progressively reduced as onset age increased, and the HR and 95% CI decreased from 1.84 (1.31–2.57) at age < 45 years to 1.28 (1.10–1.50) at age ≥65 years. The participants of our study had the exact diagnosis time of the MetS, which could mitigate the impact of underlying prevalence-incidence bias, unlike the ULSAM study; thus, the association between exposure and outcome was more reliable. In this case, it can be observed that the results of the ULSAM study may overestimate the relative risk of the MetS and CVD when compared with our study. Although there is no study similar to ours at present, Dugani et al. detected that the HR and 95% CI of the MetS with coronary heart disease (CHD) in women aged below 50 years was 6.09 (3.60–10.29) in the study exploring the association between multiple risk factors and onset age of CHD in women, and this risk decreased with the increasing onset age of CHD (23). In addition, Eddy et al. reported that among the components of MetS, the most important single factor for identifying CVD risk was hyperglycemia, with hypertension, obesity, increased triglycerides and decreased HDL following in that order (24). Indeed, existing evidence indicated that hyperglycemia and hypertension are strongly associated with the incident of CVD (25–27). Recent studies have discovered that among the major components of the MetS, young individuals with new-onset hypertension or diabetes have a higher relative risk of CVD (9–11), which indirectly supports our findings.

Consistent with the trend of MetS young onset, the incidence of CVD in young individuals (18–50 years old) also showed a steady upward trend (28, 29). However, there may be subtype differences between the MetS and growing CVD risk in young individuals. ULSAM study had found that the risk ratio of the MetS and MI at age 50 was 2.87 (1.48–4.48), lower than that of IS, being 3.55 (1.38–9.10) (8). Whereas, in our study, it was detected that the HR for the new-onset MetS and MI at age <45 years was 3.14 (1.31–7.50), higher than that of IS being 1.97 (1.30–2.98). This discrepancy between the ULSAM study result and ours may be owing to the participants in our study being Asians, and the young population was relatively younger and had the exact MetS exposure time; however, our results should be replicated among other groups.

Since our study was an observational study, it was unable to determine the mechanism of the new-onset MetS and the relative risk of CVD at different ages; however, it may be attributed to the following factors. Firstly, central obesity is dominant in the MetS, and the results of a large meta-analysis (30) demonstrated that obesity in young individuals was more susceptible to genetic susceptibility than that in the elderly population. Genetic variation associated with the MetS has also been proven to be associated with CVD (31–33); therefore, the genetic variation leading to the occurrence of MetS may directly lead to an increased risk of CVD. Secondly, the new-onset MetS has been linked to unhealthy lifestyles among young individuals, such as high-fat and high-sugar diets, smoking, drinking, and lack of exercise, which are also risk factors for CVD. Finally, most young individuals are not aware of the increased risk of CVD brought by the MetS. Compared with the elderly, young individuals have lower awareness, treatment, and control rate of hypertension, diabetes, or dyslipidemia (34–36); therefore, young new-onset MetS had not received good intervention and treatment, which might be one of the reasons for the significant increase in CVD risk.

The significance of our study is to find that new-onset MetS are associated with a higher relative risk of CVD among young individuals. Moreover, the adverse outcomes of young individuals will lead to serious loss of the labor force, longer survival time with the disease, large consumption of medical resources, and increasing economic burden of society and family. In addition, studies have demonstrated that once diabetes, hypertension, or hyperlipidemia occurs, even if blood glucose, blood pressure, or blood lipids reach the control target, there is still a residual risk of CVD (37, 38). Therefore, young individuals should be encouraged to actively quit smoking, lose weight, adopt Mediterranean diet and maintain physical exercise according to the international recommendations (39) to prevent the MetS and reduce the risk of CVD. At the same time, appropriate use of nutraceuticals is effective for MetS prevention to a certain extent (40). Park et al. (41) discovered that individuals who recovered from the MetS had a reduced relative risk of CVD than those featured with stable MetS; thus, early identification and intervention for MetS are also practically significant. The existing guidelines for the treatment of hypertension, diabetes, and dyslipidemia focus on combination medication that mitigates the CVD risk among middle-aged and elderly individuals while ignoring younger individuals (38, 42, 43). In this context, our results will provide new evidence that control and management of the young new-onset MetS should be strengthened at an early stage.

This study has several strengths. Firstly, it is the first large prospective study that explores the association between the new-onset MetS of different ages and the CVD risk. Having the exact diagnosis time of the MetS makes the association between exposure and outcome more reliable. Secondly, age ( ± 1 year) and sex-matched non-MetS controls were included, when considering and reducing the influence of age and sex on the results. In addition, the whole research population was covered by biennial physical examinations, hospital discharge registrations, and municipal social insurance institutions, which permitted us to accurately track endpoint events for all participants. Nevertheless, inevitably, this study is featured with the following limitations: firstly, the MetS is the status that is prone to change, and it is challenging to control its status or composition change; however, as a multi-component combination, the MetS can be clinically identified as a higher risk factor for CVD, and our study focused on MetS onset age when only the time of accurate exposure is required. Secondly, Polycystic ovary syndrome (PCOS) is a common risk factor of CVD in young women (44–46), but we could not determine and exclude young women with PCOS in this study, which might have potential, but minor effect on the results. Thirdly, there is a large proportion of males from North China in this study, and the generalizability is limited; thus, our result should be verified in other populations. Finally, the follow-up time in the research is relatively short; thus, a longer follow-up time should be allocated to verify the results of this study in the future.

## Conclusions

Our study demonstrates the higher relative risk of CVD among participants with young new-onset MetS, and the relative risk of CVD decreases with increasing age of MetS diagnosis. In addition, the young individuals with the new-onset MetS tend to overlook potential health hazards, which will increase the burden of CVD for forthcoming decades; therefore, relevant measures should be taken to identify the MetS early, conduct intervention, strengthen management, and reduce their CVD risk.

## Data Availability Statement

The original contributions presented in the study are included in the article/**Supplementary Material**. Further inquiries can be directed to the corresponding authors.

## Ethics Statement

The studies involving human participants were reviewed and approved by The Ethics Committee of Kailuan General Hospital (Approval Number: 2006-05). The patients/participants provided their written informed consent to participate in this study.

## Author Contributions

The study idea was designed by ZH, SW, and YC. ZH, XW, XD, ZFC, ZKC, and ZWC analyzed and interpreted the data. ZH, GC, XW, YL, WL, WF, WW, and ZCC were responsible for drafting the manuscript. The manuscript was reviewed by SW and YC. All authors have read and approved the final manuscript.

## Funding

This work has been supported by the National Natural Science Foundation of China (No. 81870312).

## Conflict of Interest

The authors declare that the research was conducted in the absence of any commercial or financial relationships that could be construed as a potential conflict of interest.

## Publisher’s Note

All claims expressed in this article are solely those of the authors and do not necessarily represent those of their affiliated organizations, or those of the publisher, the editors and the reviewers. Any product that may be evaluated in this article, or claim that may be made by its manufacturer, is not guaranteed or endorsed by the publisher.

## Abbreviations

MetS, metabolic syndrome; CVD, cardiovascular diseases; HR, hazard ratios; CI, confidence interval; WC, waist circumference; SBP, systolic blood pressure; DBP, diastolic blood pressure; HDL-C, high-density lipoprotein cholesterol; FPG, fasting plasma glucose; hs-CRP, high-sensitivity C-reactive protein; MI, myocardial infarction; IS, ischemic stroke; HS, hemorrhagic stroke.
