# Supplementary material for: Association of Age of Metabolic Syndrome Onset With Cardiovascular Diseases: The Kailuan Study
Source: Front Endocrinol (Lausanne). 2022 Mar 17;13:857985. doi: 10.3389/fendo.2022.857985 (PMC8968729; doi:10.3389/fendo.2022.857985)
Supplement: Supplementary file 1 [file DataSheet_1.docx]

**Figure S1.** Flow chart of inclusion and exclusion.

**Caption:** The flowchart of 25,125 new-onset MetS participants and 25,125 non-MetS participants included in the final analyses.


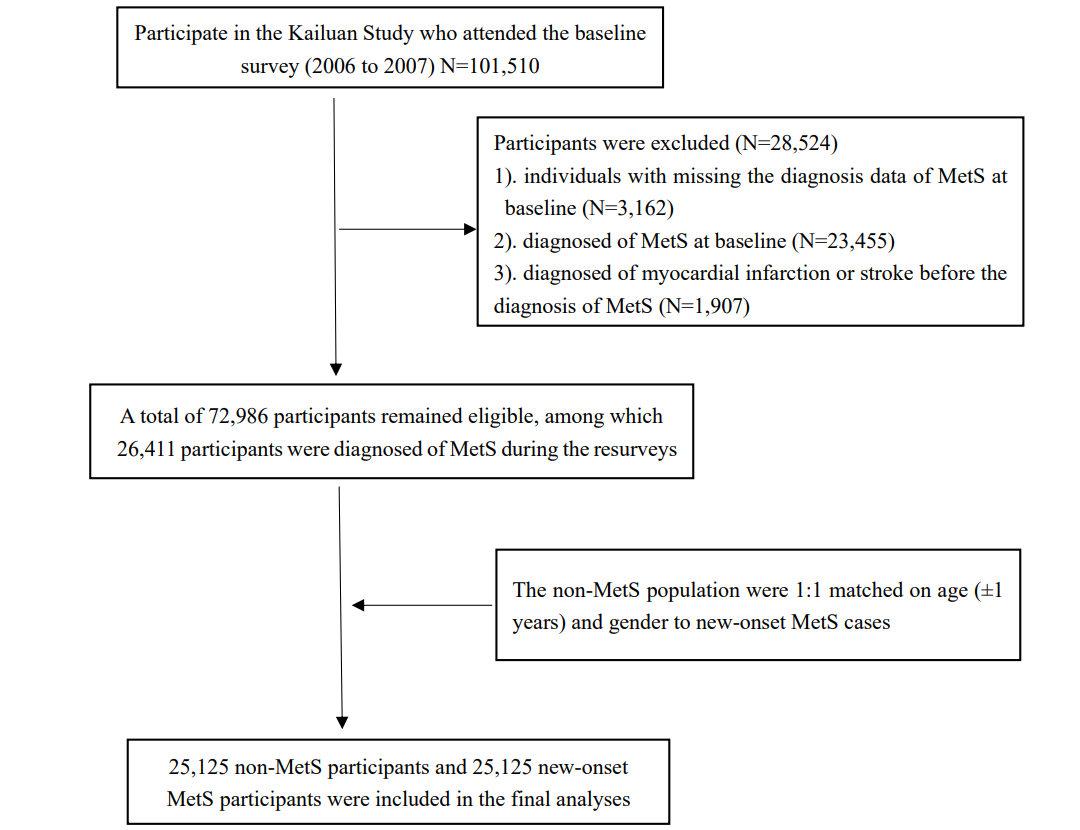


**Table S1.** Sensitivity Analysis Using the CDS, NCEP, or JIS definition of MetS.

| MetS Onset Age | Non-MetS | New-Onset MetS |
| --- | --- | --- |
| CDS definition (N = 47,658) |  |  |
| <45 Y | Ref. | 1.98(1.34-2.81) |
| 45~54 Y | Ref. | 1.94(1.63-2.32) |
| 55~64 Y | Ref. | 1.46(1.25-1.70) |
| ≥65 Y | Ref. | 1.35(1.13-1.61) |
| p for interaction |  | 0.01 |
| NCEP definition（N=37,706） |  |  |
| <45 Y | Ref. | 1.91(1.24-2.90) |
| 45~54 Y | Ref. | 1.79(1.48-2.18) |
| 55~64 Y | Ref. | 1.59(1.34-1.87) |
| ≥65 Y | Ref. | 1.51(1.25-1.83) |
| p for interaction |  | 0.37 |
| JIS definition (N = 50,198) |  |  |
| <45 Y | Ref. | 1.72(1.21-2.40) |
| 45~54 Y | Ref. | 1.77(1.50-2.14) |
| 55~64 Y | Ref. | 1.34(1.15-1.55) |
| ≥65 Y | Ref. | 1.36(1.16-1.62) |
| p for interaction |  | 0.03 |

The models were adjusted for HR, hs-CRP, ever-smokers, ever-drinkers, physical exercise, education and family history of cardiovascular disease.

**Table S2.** Sensitivity Analysis Excluding Outcome Events within the first year of Follow-up (N = 49,332)

| MetS Onset Age | Non-MetS | New-Onset MetS |
| --- | --- | --- |
| <45 Y | Ref. | 1.83(1.29-2.59) |
| 45~54 Y | Ref. | 1.72(1.45-2.03) |
| 55~64 Y | Ref. | 1.35(1.16-1.57) |
| ≥65 Y | Ref. | 1.21(1.03-1.44) |
| p for interaction |  | <0.01 |

The models were adjusted for HR, hs-CRP, ever-smokers, ever-drinkers, physical exercise, education and family history of cardiovascular disease.

**Table S3.** Sensitivity Analysis Excluding received medical treatment of Follow-up (N = 30,678)

| MetS Onset Age | Non-MetS | New-Onset MetS |
| --- | --- | --- |
| <45 Y | Ref. | 1.96(1.26-3.04) |
| 45~54 Y | Ref. | 1.66(1.33-2.06) |
| 55~64 Y | Ref. | 1.28(1.05-1.58) |
| ≥65 Y | Ref. | 1.30(1.03-1.63) |
| p for interaction |  | 0.09 |

The models were adjusted for HR, hs-CRP, ever-smokers, ever-drinkers, physical exercise, education and family history of cardiovascular disease.

**Table S4.** Competing risk analyses of cardiovascular disease among patients with New-Onset MetS versus Non-MetS across age groups.

| MetS Onset Age | Non-MetS | New-Onset MetS |
| --- | --- | --- |
| <45 Y | Ref. | 1.84(1.31-2.58) |
| 45~54 Y | Ref. | 1.67(1.43-1.96) |
| 55~64 Y | Ref. | 1.38(1.19-1.58) |
| ≥65 Y | Ref. | 1.30(1.12-1.52) |

The models were adjusted for HR, hs-CRP, ever-smokers, ever-drinkers, physical exercise, education and family history of cardiovascular disease.
